# Impact of health literacy and primary language on the decision to pursue trial of labor after prior cesarean delivery

**DOI:** 10.1186/s12884-025-07788-6

**Published:** 2025-06-03

**Authors:** Daisy Leon-Martinez, Christine Dehlendorf, Molly Zeme, W. John Boscardin, Anjali J. Kaimal, William A. Grobman, Miriam Kuppermann

**Affiliations:** 1 Department of Obstetrics, Gynecology & Reproductive Sciences, University of California—San Francisco, 490 Illinois Street, Floor 10, San Francisco, CA 94143 USA; 2 Department of Family and Community Medicine, University of California—San Francisco, 2540 23rd Street, #5702, San Francisco, CA 94110 USA; 3 Department of Epidemiology and Biostatistics, University of California—San Francisco, 3333 California Street, #220G, San Francisco, CA 94118 USA; 4 School of Medicine, University of California—San Francisco, 533 Parnassus Ave., San Francisco, CA 94143 USA; 5 Department of Obstetrics and Gynecology, University of South Florida Morsani College of Medicine, 2 Tampa General Circle, 6th Floor, Tampa, FL 33606 USA; 6 Department of Obstetrics and Gynecology, The Warren Alpert Medical School of Brown University, 101 Dudley St., Providence, RI 02905 USA

**Keywords:** Health literacy, Primary language, Trial of labor after cesarean, TOLAC, Vaginal birth after cesarean, VBAC, Decision quality, Shared decision-making, Decision self-efficacy, Decision satisfaction

## Abstract

**Background:**

Both a trial of labor after cesarean (TOLAC) and elective repeat cesarean delivery (ERCD) are reasonable choices after a cesarean delivery, with differing risks and benefits. This study explores the impact of patient health literacy and primary language on the decision to pursue a TOLAC and on decision quality.

**Methods:**

This is a secondary analysis of the Prior Cesarean Decision (PROCEED) trial, which examined the effect of a patient-centered decision support tool on rates of TOLAC and decision quality. Logistic regression was performed to estimate the association of limited health literacy (Newest Vital Sign score ≤4/6) and non-English primary language (NEPL) with TOLAC. Decision quality was assessed by calculating mean scores for decision-quality scales and using linear regression to estimate adjusted mean differences (aMD) by health literacy and NEPL.

**Results:**

Among 1455 participants, 44.6% underwent TOLAC, and 71.0% of those with a TOLAC had a vaginal birth after cesarean (VBAC). Limited health literacy was associated with lower odds of TOLAC (aOR 0.60, 95% CI [0.38, 0.93]). For decision quality, limited health literacy was associated with similar scores for decisional conflict, shared decision-making, decision self-efficacy and decision satisfaction, but lower knowledge scores (3.9 vs. 5.4; aMD -0.7, 95% CI [-1.0, -0.5]). Compared to participants whose primary language was English (*n*=1043), those with NEPL (*n*=255) had similar odds of TOLAC (aOR 1.08, 95% CI [0.69, 1.68]), but greater decisional conflict (20.9 vs. 16.7; aMD 3.9, 95% CI [1.4, 6.3]) and lower decision self-efficacy (88.6 vs. 90.9; aMD -3.3, 95% CI [-5.6, -1.1]) and decision satisfaction (4.6 vs. 4.7; aMD −0.1, 95% CI [-0.2, 0.0]).

**Conclusions:**

In this study of pregnant people with a prior cesarean and no prior VBAC, those with limited health literacy had lower odds of TOLAC and lower knowledge scores about risks and benefits of TOLAC vs. ERCD. While those with NEPL had similar odds of TOLAC, they had lower decision quality scores compared to those with those with English as a primary language. These findings indicate factors that may result in less effective counseling related to delivery options after prior cesarean and may contribute to differences in approach to delivery and decision quality.

**Supplementary Information:**

The online version contains supplementary material available at 10.1186/s12884-025-07788-6.

## Background

Ensuring high quality, evidence-based, and equitable care is critical to supporting optimal pregnancy outcomes [1]. Integral to high quality obstetric care is supporting and ensuring patient autonomy and empowering informed patient decision-making [2]. This importance has been highlighted in the context of respectful obstetric care, a proposed driver of health equity [3–5]. 

Counseling a pregnant person who has had a previous cesarean delivery about delivery approach is an especially complex process [6, 7]. Existing evidence suggests that both an elective repeat cesarean delivery (ERCD) and trial of labor after cesarean (TOLAC) are reasonable choices [8]. The American College of Obstetrics and Gynecology (ACOG) recommends that providers counsel on the risks and benefits of both, incorporating individual patient characteristics that may affect the likelihood of complications from TOLAC and ERCD [9]. The majority of known complications with both options, while potentially devastating, are exceedingly rare [8]. 

Accordingly, it is important to ground individual patient counseling in clear discussions of risks and benefits and to recognize the central role of patient values and priorities in delivery decisions. Given documented disparities in both obstetric outcomes and experiences of care by race, ethnicity and socioeconomic status, understanding the factors that affect decision-making regarding delivery approach after a prior cesarean delivery can inform efforts to ensure that care and decision making are optimized for all patients. Patient health literacy and language are two factors that are important to explore, as existing obstetric literature has demonstrated an association between these factors and engagement in meaningful shared decision-making [10–13]. Among non-obstetric multi-lingual cohorts, limited health literacy and having a non-English primary language (NEPL) have also been associated with negative health-information seeking experiences (e.g., more effort, frustration, concern about quality, and difficulty understanding information) [14] and suboptimal shared decision making [13]. 

This study aims to explore the association between limited health literacy and NEPL in pregnant people who are eligible for TOLAC and the decision to pursue TOLAC and achieve vaginal birth after cesarean (VBAC). We further aim to explore the association between limited health literacy and NEPL and patient-reported decision quality.

## Methods

This is a secondary analysis of the PROCEED trial (ClinicalTrials.gov Identifier: NCT02646423; registration date: 2015-12-15), which was conducted between January 2016-January 2019 at three academic medical centers and two community sites and examined the effect of a patient-centered decision support tool on rates of TOLAC, VBAC and decision quality. At all study sites, usual care for patients with a prior cesarean included counseling by the provider regarding delivery approach (TOLAC vs. ERCD). Details of the trial have been published previously [15]. Briefly, English- or Spanish-speaking pregnant people with one prior cesarean delivery, no prior VBAC, no known absolute contraindication to trial of labor (e.g., prior classical or T incision cesarean delivery, prior uterine surgery), and a singleton gestation between 12 weeks 0 days and 24 weeks 6 days were randomized to use or not use a decision support tool regarding delivery approach that was provided on an electronic tablet. Both groups otherwise received usual care at their clinical sites, including counseling by a clinician regarding delivery approach. Because rates of cesarean delivery have been shown to differ by the social construct of race and ethnicity [8], participant self-reported race and ethnicity were obtained. Between 34 weeks 0 days and 37 weeks 6 days of gestation, patient-reported outcomes were assessed during a telephone interview conducted by a research staff member who was unaware of group assignment. Delivery approach (TOLAC or ERCD) and mode (VBAC or repeat cesarean delivery [RCD]) were ascertained postpartum via medical record review conducted by clinician investigators, who also were unaware of the randomization group. This study was conducted in accordance with the ethical guidelines of the Declaration of Helsinki and was approved by the institutional review board at each of the participating sites. All participants provided written informed consent prior to study enrollment. The datasets used and/or analyzed during the current study are available from the corresponding author on reasonable request.

All study participants were included in the current analysis. Individuals were compared according to their level of health literacy, which was assessed during the baseline questionnaire through administration of the Newest Vital Sign (NVS) [16]—an instrument previously validated in both English and Spanish that evaluates the ability to interpret a nutrition label through six associated questions. A score of ≤4 has been used to define limited health literacy, and > 4 to define adequate health literacy [16]. Individuals were also compared according to their primary language, which was self-reported during the preliminary eligibility screening. All study instruments were available in English and Spanish and administered in the language preferred by the participant.

The primary outcome for this analysis was delivery approach (TOLAC vs. ERCD). Mode of delivery (VBAC vs. RCD) was a secondary outcome. Additional secondary outcomes included measures of decision quality (decisional conflict, knowledge, shared decision-making, decision self-efficacy, and decision satisfaction). Validated scales were administered by phone between 34 weeks 0 days and 37 weeks 0 days gestation. Decisional conflict was measured using the Decisional Conflict Scale [17, 18], a validated instrument composed of 16-items that generates an overall score and five subscale scores ranging from 0 to 100, with scores > 25 indicating clinically important decisional conflict [19]. Knowledge about TOLAC and ERCD was assessed using a modified version of an 8-item questionnaire [20]. The 9-item Shared Decision-Making Questionnaire (SDM-Q-9), a psychometrically evaluated tool, was used to evaluate shared decision-making [21, 22], and the 11-item Decision Self-efficacy Scale assessed the participants’ perceived ability to engage in the decision-making process [23]. Both the SDM-Q-9 and the Decision Self-efficacy Scale range from 0 to 100 with higher scores denoting higher degrees of shared and self-efficacious decisions respectively. Decision satisfaction was assessed using the Satisfaction with Decision Scale. This 6-item instrument was designed to measure global satisfaction with a health decision; scores range from 1 to 5, with a reported mean of 3.9 in the validation study [24]. Validated Spanish-language instruments were used when available; other instruments were forward- and back-translated by native Spanish speakers and certified interpreters.

### Statistical analyses

Demographic characteristics and prior obstetric history were compared between groups using the chi-square test for categorical variables and the Wilcoxon rank-sum test for continuous variables. Using multivariable logistic regression models, we estimated the association of limited health literacy (defined as NVS score ≤4) with TOLAC and VBAC, and we estimated the association of NEPL with TOLAC and VBAC. We estimated the association of health literacy with decision quality by calculating the mean scores for each scale by level of health literacy (limited vs. adequate) and estimated adjusted mean differences (aMD) using multivariable linear regression. A similar approach was used to estimate the association of NEPL and measures of decision quality. Informed by existing literature and clinical importance, we used a directed acyclic graph (Figure S1) to identify the minimally sufficient adjustment set of covariates: age, race, income, primary language, relationship status and recruitment site. To account for imbalance in baseline characteristics, we also adjusted for body mass index (BMI) [25], history of prior vaginal birth [25], initial inclination about intended delivery approach, and insurance. The same model was used for adjusted estimation of each secondary outcome. For TOLAC and VBAC, we elected a priori to perform interaction analyses for effect modification of health literacy by randomized treatment group, recruitment site, and primary language. In a sensitivity analysis, we handled missing data by applying multiple imputation using chained equations [26]. Statistical tests were performed with STATA version 17 (STATA Corp, College Station, TX, USA).

## Results

Of 1470 pregnant people included in the dataset of the parent trial, 15 (1.0%) were missing baseline data regarding health literacy and were excluded from this analysis, resulting in 1455 individuals in this analytic dataset (Table 1). Among these participants, 396 (27.2%) had limited health literacy, and 255 (17.5%) reported having a primary language other than English. Baseline characteristics among individuals with limited health literacy versus those with adequate health literacy differed for several characteristics (Table 1), including stronger initial preferences for ERCD among those with adequate health literacy than among those with low health literacy. Additionally, people with NEPL were more likely to have low health literacy (41.4%) compared to adequate health literacy (7.9%).

**Table 1:** Baseline demographic and clinical characteristics presented by level of health literacy

Characteristics ^a^	Limited health literacy ^f^ (*n*=396)	Adequate health literacy ^f^ (*n*=1059)	
Sociodemographic characteristics			
Age, mean (SD), y	31.9 (5.1)	34.9 (4.0)	
Race or ethnic group			
African American or Black	66 (16.7%)	49 (4.6%)	
Asian or Pacific Islander	57 (14.4%)	159 (15.0%)	
Bi- or multi-racial/ethnic	14 (3.5%)	34 (3.2%)	
Caucasian, White or European American	83 (21.0%)	716 (67.6%)	
Latina, Latin American, or Hispanic	164 (41.4%)	76 (7.2%)	
Other^b^	12 (3.0%)	25 (2.4%)	
Non-English Primary Language	164 (41.4%)	84 (7.9%)	
Opted for Spanish language interview	91 (23.0%)	12 (1.1%)	
Educational attainment			
Less than a high school degree	38 (9.6%)	1 (0.1%)	
High school graduate, GED or equivalent	88 (22.2%)	13 (1.2%)	
Some college, junior college or vocational school	87 (22.0%)	81 (7.6%)	
College graduate (BA, BS)	118 (29.8%)	432 (40.8%)	
Professional or graduate degree (MA, MS, MBA, PhD, MD, JD, etc.)	65 (16.4%)	532 (50.2%)	
Relationship status			
Married or living with partner	331 (83.6%)	1032 (97.5%)	
Significantly involved with a partner, but not living together	30 (7.6%)	17 (1.6%)	
Single/not significantly involved	35 (8.8%)	10 (0.9%)	
Yearly household income			
Under $25,000	84 (21.2%)	16 (1.5%)	
$25,000 - $50,000	99 (25.0%)	31 (2.9%)	
$50,001 - $100,000	70 (17.7%)	127 (12.0%)	
$100,001 - $200,000	67 (16.9%)	378 (35.7%)	
Over $200,000	38 (9.6%)	484 (45.7%)	
Don’t know	32 (8.1%)	15 (1.4%)	
Decline to answer	6 (1.5%)	7 (0.7%)	
Insurance			
Private insurance	194 (49.0%)	985 (93.1%)	
Public insurance	200 (50.5%)	65 (6.1%)	
Other, specify	2 (0.5%)	8 (0.8%)	
Clinical characteristics			
Recruitment site location			
UCSF	71 (17.9%)	308 (29.1%)	
Marin Community Clinic	44 (11.1%)	6 (0.6%)	
Northwestern	77 (19.4%)	369 (34.8%)	
Massachusetts General Hospital	187 (47.2%)	357 (33.7%)	
St Luke’s	17 (4.3%)	19 (1.8%)	
Pre-pregnancy BMI, median (IQR), kg/m^2^	27.4 (23.4, 32.0)	24.4 (21.7, 28.1)	
Prior vaginal delivery	43 (11.0%)	42 (4.0%)	
Indication for primary cesarean delivery			
Arrest disorder ^c^	163 (42.4%)	472 (45.2%)	
Fetal indication ^d^	199 (51.8%)	504 (48.3%)	
Maternal clinical indication ^e^	18 (4.7%)	60 (5.7%)	
Maternal request	4 (1.0%)	8 (0.8%)	
Initial delivery approach inclination			
Definitely a repeat c-section	87 (22.0%)	208 (19.6%)	
Probably a repeat c-section	54 (13.6%)	235 (22.2%)	
I’m not sure/I don’t know	42 (10.6%)	140 (13.2%)	
Probably trying to have a vaginal delivery	84 (21.2%)	195 (18.4%)	
Definitely trying to have a vaginal delivery	129 (32.6%)	281 (26.5%)	

^a^ Data reported as n(%) of participants unless otherwise indicated

^b^ Includes Middle Eastern, North African, South Asian, Caribbean, Turkish, non-Latina South American, and Jewish (participant self-report)

^c^ Includes cephalopelvic disproportion, arrest of dilation, arrest of descent, active phase arrest, and failed induction

^d^ Includes non-reassuring fetal status, oligohydramnios, breech presentation, multiple gestations, macrosomia, prior shoulder dystocia, and abruption

^e^ Includes preeclampsia, placenta or vasa previa, and other medical conditions

^f^ Limited health literacy (NVS score ≤ 4/6), adequate health literacy (NVS score ≥ 5/6)

Overall, 44.6% of participants underwent TOLAC (43.7% of participants with limited health literacy, 45.0% of participants with adequate health literacy, *p* = 0.65) (Table 2). Compared to individuals with adequate health literacy, after adjusting for covariates (primary language, age, income, insurance, race, BMI, relationship status, history of prior vaginal delivery, desired delivery approach at enrollment, enrollment site), those with limited health literacy had lower odds of undergoing TOLAC (aOR 0.60, 95% CI [0.38, 0.93]). However, among participants who underwent TOLAC, those with limited health literacy had similar odds of achieving VBAC as those with adequate health literacy (aOR 1.10, 95% CI [0.58, 2.10]) (Table 2). When comparing the decision to pursue TOLAC by participants’ primary language, those with NEPL were more likely to undergo TOLAC (50.4% vs. 43.0%, *p* = 0.04), but after adjustment for covariates, their odds of pursuing TOLAC (aOR 1.08, 95% CI [0.69, 1.68]) were similar. Those who did pursue TOLAC, however, had a modestly increased odds of VBAC (aOR 1.95, 95% CI [1.01, 3.80]), compared to those who use English as their primary language (Table 2).

**Table 2:** Primary and secondary clinical outcomes, multivariable analysis

	Limited health literacy (*n*=396) ^a, b^	Adequate health literacy (*n*=1059) ^a, b^	Adjusted odds ratio ^c^ (95% CI)	*p* value
**Primary Outcome (delivery approach)**				
Trial of labor after cesarean delivery	172/394 (43.65)	473/1051 (45.00)	0.60 (0.38, 0.93)	0.02
**Secondary Outcome (mode of delivery)**				
Vaginal birth after cesarean delivery for entire group	112/394 (28.43)	346/1051 (32.92)	0.74 (0.48, 1.15)	0.18
Vaginal birth after cesarean delivery among those that underwent TOLAC	112/172 (65.12)	346/473 (73.15)	1.10 (0.58, 2.10)	0.76
	**Non-English Primary Language (** ***n*** ** = 255)** ^**a**^	**English Primary Language (** ***n*** ** = 1043)** ^**a**^	**Adjusted odds ratio** ^**d**^ **(95% CI)**	***p*** **value**
**Primary Outcome (delivery approach)**				
Trial of labor after cesarean delivery	124/246 (50.41)	443/1030 (43.01)	1.08 (0.69, 1.68)	0.74
**Secondary Outcome (mode of delivery)**				
Vaginal birth after cesarean delivery for entire group	91/246 (36.99)	311/1030 (30.19)	1.40 (0.91, 2.17)	0.13
Vaginal birth after cesarean delivery among those that underwent TOLAC	91/124 (73.39)	311/443 (70.20)	1.95 (1.01, 3.80)	0.05

^a^ Data presented as n/total (%)

^b^ Limited health literacy (NVS score ≤ 4/6), adequate health literacy (NVS score > 4/6)

^c^ Adjusted for participant primary language, age, income, insurance, race, BMI, relationship status, history of prior vaginal delivery, desired delivery approach at enrollment, enrollment site

^d^ Adjusted for participant health literacy, age, income, insurance, race, BMI, relationship status, history of prior vaginal delivery, desired delivery approach at enrollment, enrollment site

Decision quality measures varied by level of health literacy (Table 3). For example, knowledge about the risks and benefits of TOLAC and ERCD was statistically significantly lower for participants with limited health literacy compared to those with adequate health literacy (3.9 vs. 5.4; adjusted mean difference -0.7, 95% CI [-1.0, -0.5]). However, decisional conflict scores were statistically similar for those with limited and adequate health literacy (17.7 vs. 17.2; adjusted mean difference 0.52, 95% CI [ -1.82, 2.86]). Similarly, shared decision-making, decision self-efficacy, and decision satisfaction scores did not differ by level of health literacy (Table 3) [21–24]. 

**Table 3:** Participant-Reported decision quality outcomes^a^

	Mean (SD) [No.]			
	Limited health literacy ^b^ (*n*=396)	Adequate health literacy ^b^ (*n*=1059)	Adjusted mean difference (95% CI) ^c^ *	*p*-value
Knowledge ^d^	3.87 (1.83) [345]	5.41 (1.49) [1001]	-0.74 (-1.01, -0.47)	< 0.001
Decisional conflict ^e^	17.70 (12.11) [339]	17.16 (13.83) [997]	0.52 (-1.82, 2.86)	0.662
Uncertainty subscale	22.08 (18.00) [343]	25.71 (22.89) [1001]	1.56 (-2.11, 5.23)	0.404
Informed subscale	15.50 (12.75) [344]	12.98 (12.89) [1000]	-0.83 (-3.12, 1.47)	0.48
Values clarity subscale	18.37 (14.09) [342]	16.81 (16.14) [1000]	0.71 (-2.08, 3.49)	0.619
Support subscale	15.37 (15.00) [341]	12.19 (15.61) [1001]	0.63 (-2.11, 3.37)	0.651
Effective decision subscale	23.16 (19.14) [340]	23.76 (20.97) [999]	1.21 (-2.34, 4.76)	0.504
Shared decision-making ^f^	76.86 (14.26) [338]	73.88 (15.69) [987]	1.37 (-1.32, 4.07)	0.316
Decision self-efficacy ^g^	91.09 (11.54) [338]	90.39 (12.34) [993]	-0.82 (-2.99, 1.36)	0.462
Decision satisfaction ^h^	4.72 (0.51) [344]	4.61 (0.58) [999]	0.04 (-0.05, 0.13)	0.395
	**Non-English Primary Language (** ***n*** **=255)**	**English Primary Language (** ***n*** **=1043)**	**Adjusted mean difference (95% CI)** ^**c**^ **	***p*** **-value**
Knowledge ^d^	4.27 (1.96) [222]	5.19 (1.61) [965]	0.09 (-0.18, 0.37)	0.515
Decisional conflict ^e^	20.94 (13.11) [218]	16.72 (13.46) [961]	3.86 (1.44, 6.28)	0.002
Uncertainty subscale	26.89 (20.36) [220]	24.43 (22.10) [965]	4.76 (0.98, 8.54)	0.014
Informed subscale	17.35 (12.60) [220]	13.06 (13.02) [965]	2.03 (-0.34, 4.40)	0.093
Values clarity subscale	21.14 (16.13) [220]	16.57 (15.67) [963]	3.30 (0.43, 6.17)	0.024
Support subscale	18.14 (15.71) [220]	11.91 (15.23) [964]	5.02 (2.20, 7.85)	0.001
Effective decision subscale	27.93 (20.38) [219]	23.23 (20.50) [962]	5.44 (1.77, 9.10)	0.004
Shared decision-making ^f^	75.01 (13.31) [216]	74.44 (15.77) [954]	-0.78 (-3.57, 2.00)	0.581
Decision self-efficacy ^g^	88.57 (12.34) [218]	90.91 (12.02) [957]	-3.33 (-5.58, -1.08)	0.004
Decision satisfaction ^h^	4.63 (0.57) [221]	4.67 (0.49) [963]	-0.12 (-0.21, -0.03)	0.010

* adjusted for primary language, enrollment site, delivery approach now, age, prior vaginal delivery, income, insurance, race, BMI, relationship status

** adjusted for health literacy, enrollment site, delivery approach now, age, prior vaginal delivery, income, insurance, race, BMI, relationship status

^a^ Decision quality is defined as knowledge about trial of labor after cesarean delivery and elective repeat cesarean delivery, decisional conflict (overall and by subscale), shared decision-making regarding delivery approach, decision self-efficacy, and decision satisfaction

^b^ Limited health literacy (NVS score≤4/6), adequate health literacy (NVS score>4/6)

^c^ Intervention effects are quantified as differences between groups in the mean value for each scale, estimated using linear regression models adjusted for the variables denoted by (*) and (**) above

^d^ Knowledge scores range from 0 to 8, with higher scores indicating greater knowledge

^e^ Decisional Conflict Scale scores range from 0 to 100, with higher scores indicating feeling more conflict, less informed, less certainty, less clear about personal values for benefits and risks/adverse effects, less supported in decision-making, and more that the decision was bad

^f^ Shared Decision-Making Questionnaire scores range from 0 to 100, with higher scores indicating more shared decision-making

^g^ Decision Self-efficacy Scale scores range from 0 to 100, with higher scores indicating greater self-efficacy

^h^ Satisfaction With Decision Scale scores range from 0 to 5, with higher scores indicating more satisfaction

When measures of decision quality were assessed in relation to participant primary language, having a NEPL was associated with measures of lower decision quality (Table 3). Specifically, NEPL compared to English primary language was associated with statistically significantly higher decisional conflict (20.9 vs. 16.7; adjusted mean difference 3.9, 95% CI [1.4, 6.3]). Similarly, NEPL was associated with statistically significantly lower decision self-efficacy (88.6 vs. 90.9; adjusted mean difference -3.3, 95% CI [-5.6, -1.1]) and lower decision satisfaction (4.6 vs. 4.7; adjusted mean difference -0.1, 95% CI [-0.2, 0.0]). Shared decision-making was similar between those with NEPL compared to those with English as a primary language (75.0 vs. 74.4; adjusted mean difference -0.8, 95% CI [-3.6, 2.0]). Knowledge about delivery approach also did not statistically differ by language (4.3 vs. 5.2; adjusted mean difference 0.1, 95% CI [-0.2, 0.4]).

When evaluating the outcome of TOLAC, prespecified interaction analyses showed no statistically significant effect modification between health literacy and primary language (Table S1), treatment group, or recruitment site (*p*≥0.10 for all tests for interaction). Results of the analyses using multiple imputation to address missing data did not greatly differ from those obtained in the complete case analyses.

## Discussion

Among pregnant people with one prior cesarean delivery and no prior VBAC, having limited health literacy was associated with 40% lower odds of pursuing TOLAC and with having less knowledge about the clinical risks and benefits of choosing a TOLAC or ERCD compared to those with adequate health literacy. The difference between unadjusted and adjusted results by health literacy were influenced by initial delivery preference, indicating that those with adequate health literacy were more likely to shift their decision about delivery approach towards TOLAC in response to counseling. Additionally, when making their decision about delivery approach, people with NEPL experienced higher levels of decisional conflict and reported lower self-efficacy and lower decisional satisfaction compared to those whose primary language was English. These data suggest that candidates for TOLAC who have lower health literacy or a primary language other than English are less likely to be prepared to make an informed and high-quality decision about their delivery plan.

Our results align with growing literature that shows an association between limited health literacy, patient primary language, and poor-quality outcomes among pregnant people. In a study of Latinx-identifying pregnant people, participants with Spanish preference demonstrated a lower comprehension of topics related to informed consent for common procedures during labor and delivery [27]. Similarly, a study of prenatal genetic testing uptake showed that Spanish-speaking pregnant people were less likely to recall prenatal diagnostic testing discussions with their healthcare providers, again raising concerns about the informed consent process [28]. In the context of prenatal genetic screening, individuals with low numeracy, language barriers, or a preference for provider-driven decision were also less likely to have preference concordant decision-making [29]. 

While there are many hospital-level [30, 31] and system-level [32, 33] barriers to VBAC, this work highlights that even for patients who receive care from providers who offer VBAC, there are barriers at the level of the patient-provider interaction that may preclude patients from making a high-quality decision. Taken together, these data highlight the importance of creating a patient counseling process that accounts for factors that can impact comprehension and patient decision quality, including health literacy and NEPL. Such processes may include language-concordant childbirth education [34, 35] and the incorporation of patient advocates (e.g., patient navigators [36], community health workers [37], and doulas [38, 39]), whose presence has resulted in improvement in obstetric outcomes for patients with NEPL.

Ultimately, to achieve effective, equitable, person-centered counseling, it is critical that interventions to improve patient-provider communication are informed by the greater social context. Over 8% of the US population, or greater than 25 million people, have limited English proficiency (LEP) [40]. Most (81%) are foreign-born, and nearly half (46%) have less than a high school diploma [41]. Importantly, many are less acculturated to U.S. health practices, including practices of informed consent and shared decision making. Views about how patients and their families are involved in decision making are also variable and culturally informed. Thus, innovative strategies that target multifaceted barriers (e.g., literacy, language, and cultural) are needed to address disparities in decision quality. Community engaged research, which generates bi-directional knowledge and communication between research institutions and the community to create research partnerships may be a means to address these barriers to care [42] to co-create interventions that address challenges that are meaningful to the community and effective in addressing shortcomings in decision quality. Future work is needed to develop and evaluate such interventions.

The study has many strengths, including the recruitment of patients from diverse clinical sites (academic quaternary care centers, community hospitals) with varying annual delivery volumes, neonatal care capacity, and proportions of Medicaid-insured patients. All these characteristics have been shown to influence hospital utilization and success rate of TOLAC [43]. Thus, the diversity in recruitment sites increases the generalizability of our findings. However, at the individual participant level, there are some limitations, including that study participants were more likely to elect to undergo a TOLAC (45.6%) compared to the national level (22%). This may reflect selection bias such that individuals who desire a TOLAC may be more likely to enroll in a clinical trial about decision-making regarding TOLAC. Participants were also older, had greater educational attainment, and had higher reported incomes than the general population of pregnant people. Thus, our results may represent a more informed population with greater access to resources. As a result, we may be underestimating the association of limited health literacy and the decreased likelihood of undergoing TOLAC as compared to the general US population. This underestimation may be compounded by existing racial and ethnic disparities in access to TOLAC/VBAC-supportive providers [44–46]. Additionally, this study was only conducted in English and Spanish. Therefore, our results may not reflect the counseling needs of pregnant people who have a primary language other than English or Spanish.

## Conclusions

Understanding the factors associated with differences in TOLAC is important to optimize patient decision making, ensure respectful care, and address disparities in patient experience of obstetric care and maternal morbidity and mortality. This analysis importantly identified that individuals with limited health literacy and a NEPL have lower quality decision-making regarding delivery approach, individuals with limited health literacy have lower odds of pursuing TOLAC, and individuals with a NEPL who undertake a TOLAC have greater odds of having a VBAC. Novel interventions are needed to provide competent care to those patients facing structural barriers to high-quality decision-making.

## Electronic supplementary material

Below is the link to the electronic supplementary material.

Supplementary Material 1

## Data Availability

The datasets used and/or analyzed during the current study are available from the corresponding author on reasonable request.

## Abbreviations

ACOG: American College of Obstetrics and Gynecology
aMD: Adjusted mean differences
aOR: Adjusted odds ratio
BMI: Body mass index
CD: Cesarean delivery
CI: Confidence interval
ERCD: Elective repeat cesarean delivery
LEP: Limited English proficiency
NEPL: Non-English primary language
NVS: Newest Vital Sign
PROCEED: Prior Cesarean Decision
RCD: Repeat cesarean delivery
SDM-Q-9: Shared Decision-Making Questionnaire
TOLAC: Trial of labor after cesarean
VBAC: Vaginal birth after cesarean
