# Supplementary material for: Impact of health literacy and primary language on the decision to pursue trial of labor after prior cesarean delivery
Source: BMC Pregnancy Childbirth. 2025 Jun 3;25:648. doi: 10.1186/s12884-025-07788-6 (PMC12131817; doi:10.1186/s12884-025-07788-6)

**Supplementary Materials**

**Table S1. Unadjusted odds of TOLAC for limited health literacy compared to adequate health literacy after stratifying by primary language**

| **Non-English primary language** | | |  |  | **English primary language** | | |  |  |  |
| --- | --- | --- | --- | --- | --- | --- | --- | --- | --- | --- |
|  |  | TOLAC | |  |  |  | TOLAC | |  |  |
|  |  | yes | no | Total |  |  | yes | no | Total |  |
| Health literacy | Limited n (%) | 76 (46.3) | 88 (53.7) | 164 (100) | Health literacy | Limited n (%) | 80 (41.5) | 113 (58.5) | 193 (100) |  |
|  | Adequate n (%) | 48 (58.5) | 34 (41.5) | 82 (100) |  | Adequate n (%) | 363 (43.4) | 474 (56.6) | 837 (100) |  |
|  | Total | 124 | 122 | 246 |  | Total | 443 | 587 | 1030 |  |
| TOLAC by health literacy: | | | OR 0.61 (0.36–1.04) | | OR 0.92 | (0.67–1.27) | | **Test of Homogeneity (M-H):** | | ***p* = 0.19** |

**Table S2. Adjusted odds of TOLAC for those with limited health literacy compared to adequate health literacy after adjusting for important baseline imbalances (sensitivity analyses)**

|  | **Adjusted odds ratio (95% CI)** | ***p* value** |
| --- | --- | --- |
| complete adjustment set ^a^ | 0.60 (0.38, 0.93) | 0.021 |
| excluding BMI | 0.60 (0.40, 0.92) | 0.019 |
| excluding insurance | 0.61 (0.39, 0.93) | 0.023 |
| excluding prior vaginal delivery | 0.61 (0.39, 0.93) | 0.023 |
| excluding BMI, insurance, and prior vaginal delivery | 0.62 (0.40, 0.94) | 0.024 |
| excluding desired delivery approach at enrollment | 0.76 (0.53, 1.10) | 0.146 |

^a^ primary language, age, income, insurance, race, BMI, relationship status, history of prior vaginal delivery, desired delivery approach at enrollment, enrollment site

**Figures**

**Figure S1. Directed acyclic graph**


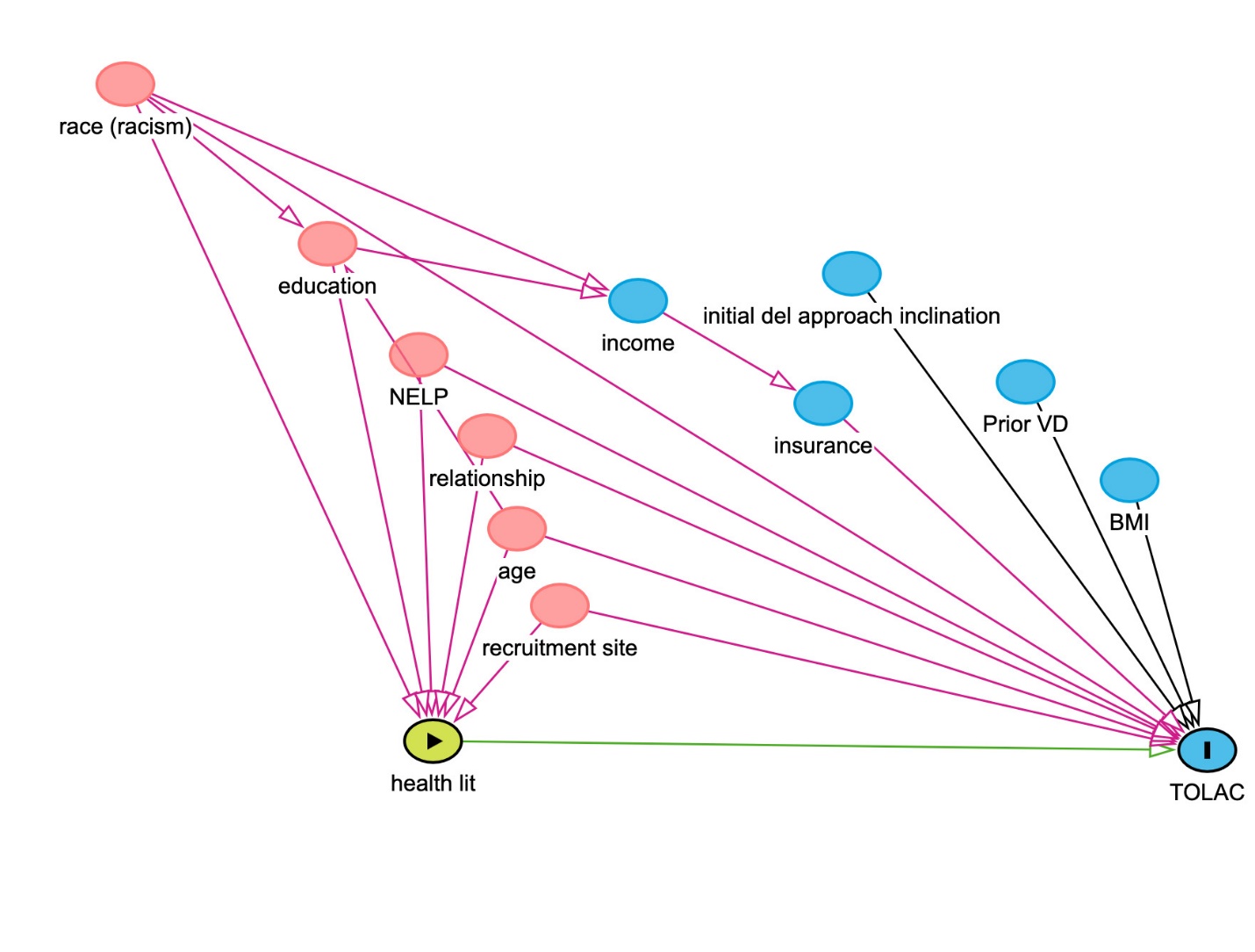

Supplement: Supplementary file 1 — Supplementary Material 1 [file 12884_2025_7788_MOESM1_ESM.docx]
